# Association of Age and Neurological Severity at Intensive Care Unit Admission With Driving Resumption Within 30 Days of Stroke: A Single-Center Historical Cohort Study

**DOI:** 10.7759/cureus.68800

**Published:** 2024-09-06

**Authors:** Chinatsu Morimatsu, Tasuku Sotokawa, Akio Kikuchi

**Affiliations:** 1 Department of Occupational Therapy, Suiseikai Kajikawa Hospital, Hiroshima, JPN; 2 Graduate School of Health Sciences, Yamagata Prefectural University of Health Sciences, Yamagata, JPN; 3 Department of Occupational Therapy, Faculty of Health Sciences, Yamagata Prefectural University of Health Sciences, Yamagata, JPN

**Keywords:** cerebral hemorrhage, stroke, driving resumption assessment, driving resumption, driving ability, nihss-national institutes of health stroke scale, post-stroke rehabilitation, acute phase, acute cerebral infarction, car driving

## Abstract

Objectives

Guidelines in several countries recommend against driving soon after a stroke; however, some patients resume driving within one month after onset. This study aimed to examine the relationship between neurological and social background factors at intensive care unit (ICU) admission and resumption of motor vehicle driving within 30 days of the first acute stroke/cerebral hemorrhage.

Materials and methods

Data were extracted from medical records of a single center linked to the National Cerebral and Cardiovascular Center Administration Office for Stroke Data Bank in Japan. The data included age, sex, Japan Coma Scale (JCS), National Institutes of Health Stroke Scale (NIHSS), employment status, family situation, and outcomes of driving resumption in patients with a valid driving license transported to the ICU within 24 hours of stroke onset. Time-to-event analysis was used to explore the associations between these factors and driving resumption, with data censored 30 days from onset.

Results

In total, 239 patients had complete medical records, of whom 66 resumed driving. A multivariate Cox proportional hazards analysis showed that fewer patients aged ≥65 years resumed driving than those aged <65 years (hazard ratio 0.46; 95% confidence interval: 0.25-0.84; p=0.009). Patients with NIHSS scores ≥5 and JCS scores ≥1 were also less likely to resume driving compared with those with scores <5 (0.22; 0.08-0.56; p=0.008) and 0 (0.13; 0.04-0.37; p<0.001), respectively.

Conclusions

Age, NIHSS score, and JCS score at ICU admission are independently associated with the likelihood of resuming driving within 30 days of stroke onset. These findings may aid with the provision of support and education to facilitate the efficient resumption of driving after an acute event.

## Introduction

Driving is a crucial mode of transportation for daily life. Resuming driving is associated with various subsequent health considerations for patients with stroke. Driving resumption in patients with stroke positively impacts social participation and health [1]. Conversely, discontinuing driving leads to negative outcomes, including depressive symptoms [2] and reduced social participation [3-5]. Additionally, research has shown a relationship between driving resumption and improved health-related quality of life, although this relationship is also influenced by other factors such as returning to work [6]. Furthermore, rehabilitation for driving resumption requires consideration of both road safety and patient health [7], because stroke-related symptoms may increase the risk of traffic accidents [8].

Several countries have rehabilitation guidelines for driving resumption, but differences exist in the timing across countries and medical conditions [9-11]. The Guidelines on Driving of Motor Vehicles by Persons with Stroke or Traumatic Brain Injury, published by the Japanese Association of Rehabilitation Medicine [12], do not specify a driving prohibition period after illness onset. Instead, they recommend submitting a medical certificate to the Public Safety Commission and following necessary procedures; however, whether to follow these procedures is up to the individual. In the United Kingdom, after a stroke or transient ischemic attack (TIA), patients are recommended to refrain from driving for one month or three months for multiple TIAs. In Australia, they are recommended to refrain from driving for four weeks after a stroke and two weeks after a TIA. In Ontario, Canada, patients with untreated cerebral aneurysms are prohibited from driving, while those with surgically treated aneurysms must wait three months. Overall, considering these guidelines, resumption of driving is not recommended for one month after stroke; however, some patients with acute stroke (30-40%) resume driving within this period [13-15]. Individuals who resume driving against medical guidelines reportedly do so because of social pressures, such as returning to work [14,16]. Additionally, some patients in acute-care hospitals lack medical records documenting their return to driving [17] and may not receive routine education on driving resumption [18].

Patients returning to work within one month of stroke onset are likely to consider driving if they have mild symptoms and were already independent in activities of daily living during hospitalization. In Japan, a study using the modified Rankin Scale (mRS) found that nearly half of patients with acute stroke at discharge from the hospital had mild symptoms [19]. Early driving resumption may be considered in these specific patient subpopulations; however, their neurological characteristics at onset are unknown, and few studies have been conducted on such patients.

We aimed to investigate the association between neurological characteristics and social background factors with motor vehicle driving resumption within 30 days after the onset of the first acute stroke (cerebral infarction or hemorrhage). The study's significance lies in the potential rationalization of patient education and motor vehicle driving assessments, if the characteristics of patients who are likely to resume driving early are known.

## Materials and methods

Study design

This was a single-center, registry-based, historical cohort study.

Participants and setting

We enrolled patients who were admitted to our specialized stroke hospital registered in the Japan Stroke Data Bank (JSDB), between January 1, 2021, and January 1, 2023, for their first-ever cerebral infarction or hemorrhage. Patients with subarachnoid hemorrhage, TIA, recurrent stroke, or cerebral hemorrhage, those unwilling to participate, those without a driving license, those with unknown outcomes, and those who were driving before the illness but not seeking a medical certificate were excluded. Patients with subarachnoid hemorrhage were excluded due to immediate surgery needs and prolonged hospital stay hindering discharge within 30 days. The study was approved by the Ethical Review Committee of Suiseikai Kajikawa Hospital (approval number: 202303) and was conducted on an opt-out basis.

Outcome/event

The study outcome was defined as the resumption of driving within 30 days of onset. The observation start date was defined as the onset date, and the end date was defined as the number of days between the onset and issuance of the medical certificate for submission to the Public Safety Commission or between the onset and the decision by the doctor to allow or disallow driving. This definition was determined based on information from medical records and other sources. Each prefectural license center determined the outcome based on the medical certificate, containing information on the ability to drive, provided by the attending physicians, who were neurosurgeons and neurologists. The medical certificate was written by the attending physician based on the results of physical function tests, observation of daily life situations, neuropsychological tests, and driving simulator (DS) evaluations conducted by the occupational therapist and physiotherapist in charge. Driving assessments of the participants were conducted considering a low risk of recurrent stroke, dementia, alcohol or drug addiction, psychosis with symptoms of hallucination, illness-causing impaired consciousness or impaired movement due to seizures, blindness, or any other condition that would be considered a punishment under the Japanese Road Traffic Law, such as revocation, suspension, or refusal of driving license.

Measurements

Variables comprised baseline characteristics, including neurological features at the time of transport and other relevant information collected from the institutional database and medical records, including sex, diagnosis (first-ever cerebral infarction, first-ever cerebral hemorrhage), age, National Institutes of Health Stroke Scale (NIHSS) and Japan Coma Scale (JCS) on admission day, and the number of days from the onset of symptoms to the date of driving resumption (the date of issuance of medical certificate for submission to the Public Safety Commission or of the decision to allow or disallow driving).

NIHSS is a widely used tool for assessing neurological deficits in patients with stroke. NIHSS consists of 15 items, including assessments of the level of consciousness, speech, language, cognition, inattention, visual field abnormalities, motor strength, sensory impairment, and ataxia. Patients are rated on a scale of 0-2, 0-3, or 0-4 points for each item, resulting in a total score of 0-42 points. Higher scores indicate increased severity [20].

JCS is a widely used method among healthcare professionals in Japan, including paramedics, for assessing the level of consciousness during the acute phase of stroke or head injury owing to its simplicity [21]. JCS scores range from 0 to 300 points and categorize consciousness levels as follows: 0 points for being fully awake and alert, 1-3 for being awake without any stimulus, 10-30 for being aroused but reverting to the previous state after stimulation cessation, and 100-300 for being unable to be aroused with any forceful mechanical stimulation. A JCS score of 1 denotes a state of nearly clear consciousness, where the patient maintains an open eye position, exhibits no disorientation, and is capable of following commands. Despite these signs of responsiveness, the patient still experiences a mild disturbance of consciousness.

Social background factors were recorded, including whether the participants had unpaid or paid work [14] and whether they lived with family members. Medical records of patients admitted to the hospital from outside their homes, such as those who suffered a stroke while being treated for another medical condition, were examined to determine whether they had a family member living with them prior to admission.

Statistical analysis

We performed comparative analyses for each variable to assess differences between patients who resumed driving within 30 days of onset and those who did not. Individuals aged ≥65 years were considered elderly according to the World Health Organization (WHO) definition. NIHSS score was analyzed and categorized into three categories, 0 (no symptoms), 1-4 (mild illness), and ≥5 (moderate or severe), based on a previous report [22]. JCS score was classified into two categories: JCS=0 and JCS≥1. Furthermore, we performed a comparative analysis after converting JCS to the Glasgow Coma Scale (GCS) [21].

Kaplan-Meier curves were used to assess the event rate of driving resumption based on different levels of each factor, and their differences were assessed through log-rank tests. The observation period lasted 30 days. The relevance of sex, age (≥65/<65 years), diagnosis (stroke/cerebral hemorrhage), NIHSS (0 points/1-4 points/≥5 points), JCS (JCS=0 or ≥1), presence of cohabiting family members (living alone/with others), employment status (employed/not employed), and number of employed individuals was assessed using adjusted/unadjusted Cox proportional hazards models. Statistical analyses were performed using R4.2.2 statistical software (R Foundation, Vienna, Austria), and the statistical significance level was set at 5%.

## Results

**Figure 1:**
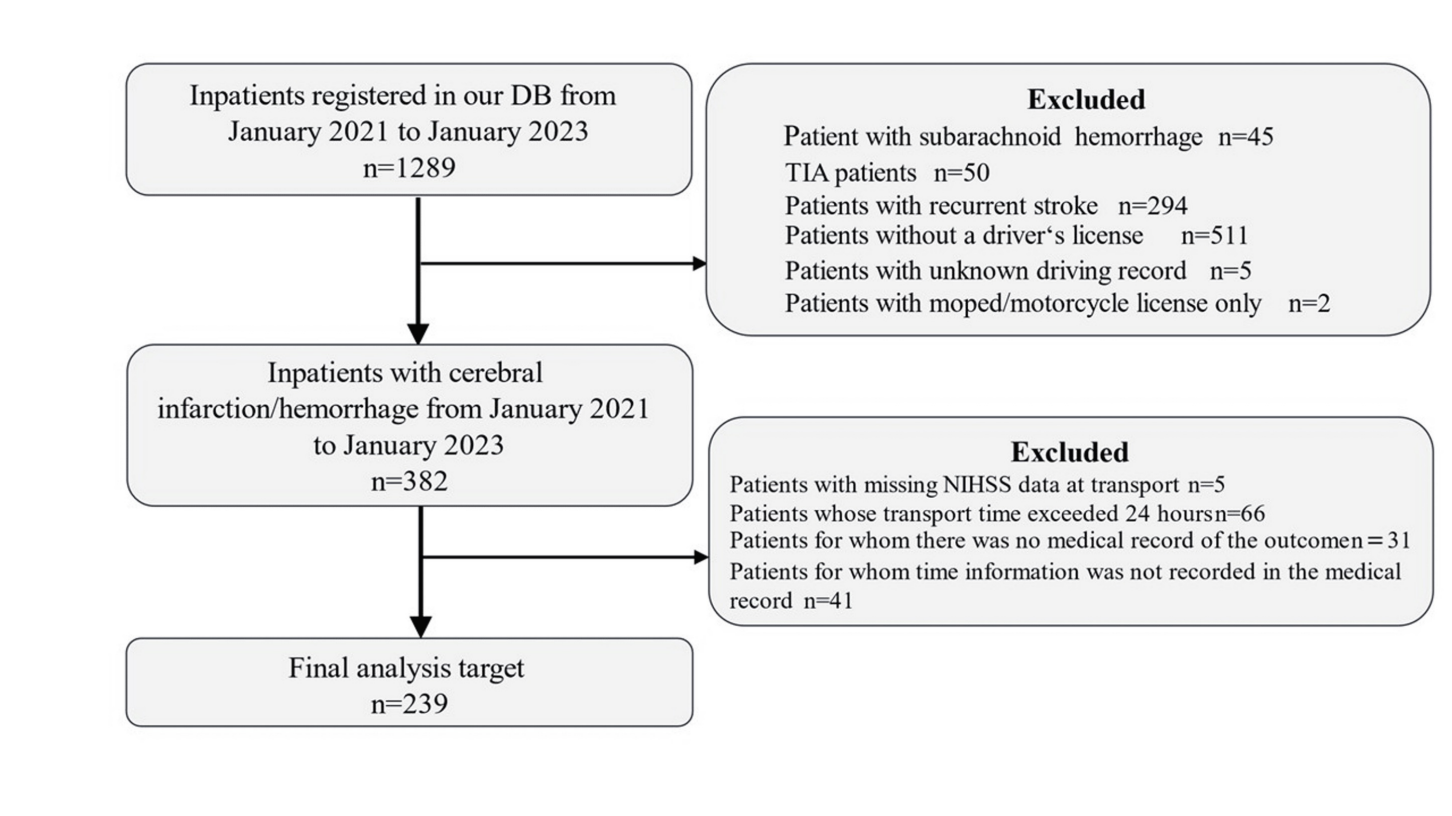
Patient selection flowchart. NIHSS: National Institutes of Health Stroke Scale; TIA: transient ischemic attack

Among the 1,289 patients admitted to the hospital during the study period, 239 were transported to the intensive care unit (ICU) within 24 hours of the onset of the first-ever stroke or cerebral hemorrhage (Figure 1).

Differences between the driving resumption group (n=66) and the non-resumption group (n=173) are presented in Table 1. There were no significant differences between the groups in terms of sex, but patients in the non-resumption group were significantly older (p<0.001). The frequency of cerebral infarction and cerebral hemorrhage was significantly different between the groups (p=0.036), and the JCS=0 and JCS≥1 categories showed significant differences in frequency (p<0.001). There were also significant differences in terms of family situation (living alone/living together; p=0.045) and employment status (p=0.003). When the JCS scores were converted to GCS, both subcategories (JCS=0 and JCS=1) were included in GCS=15, which is considered the mildest. Thus, all 66 patients in the driving resumption group were classified as GCS=15 (Table 1).

**Table 1:** Comparison of baseline characteristics between the resumed driving group and non-resumed driving group within 30 days from the onset of stroke. ^1^n (%), mean (standard deviation); ^2^Pearson's chi-squared test; ^3^Wilcoxon rank-sum test NIHSS: National Institutes of Health Stroke Scale; JCS: Japan Coma Scale; GCS: Glasgow Coma Scale

Characteristic	Overall, N=239^1^	Fit to drive, N=66^1^	Unfit to drive, N=173^1^	P-value
Sex				0.8^2^
Male	174 (73%)	49 (74%)	125 (72%)	
Female	65 (27%)	17 (26%)	48 (28%)	
Age (years)	65 (12)	61 (12)	66 (11)	<0.001^3^
Diagnosis				0.036^2^
Brain hemorrhage	67 (28%)	12 (18%)	55 (32%)	
Brain infarction	172 (72%)	54 (82%)	118 (68%)	
Total NIHSS	6 (7)	2 (3)	8 (7)	<0.001^3^
JCS				<0.001^2^
0	153 (64%)	62 (94%)	91 (53%)	
≥1	86 (36%)	4 (6.1%)	82 (47%)	
GCS				<0.001^2^
≤14	49 (21%)	0 (0%)	49 (28%)	
15	190 (79%)	66 (100%)	124 (72%)	
Family situation				0.045^2^
Living alone	45 (19%)	7 (11%)	38 (22%)	
Living together	194 (81%)	59 (89%)	135 (78%)	
Employment				0.003^2^
Employed	149 (62%)	51 (77%)	98 (57%)	
Not employed	90 (38%)	15 (23%)	75 (43%)	

**Figure 2:**
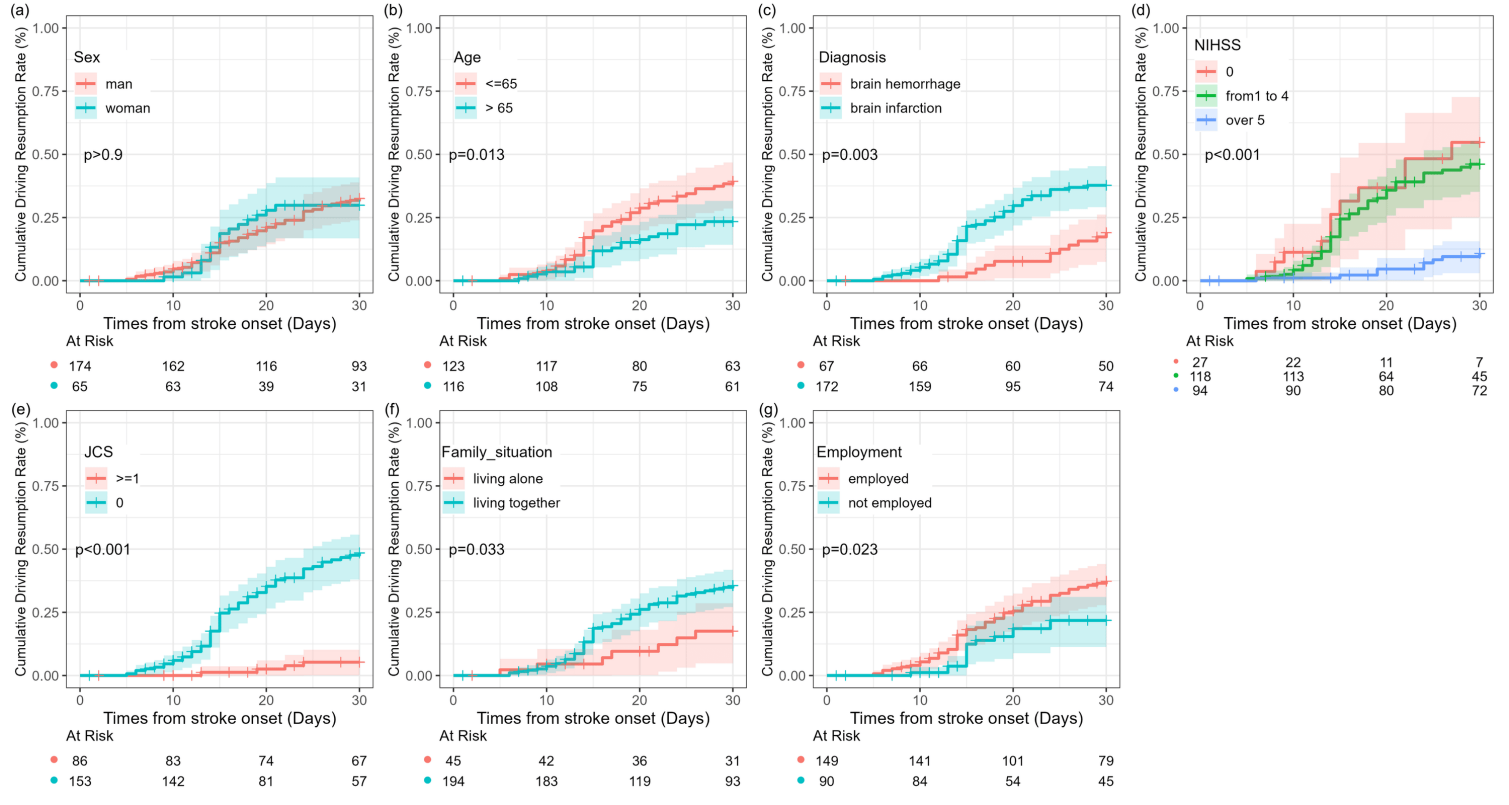
Kaplan-Meier curves and log-rank tests for cumulative driving resumption rates across multiple group factors. The Kaplan-Meier curves allow for the comparison of cumulative driving resumption rates according to (a) sex, (b) age category (≥65 or <65 years), (c) diagnosis (brain hemorrhage or brain infarction), (d) score of NIHSS (0, 1-4, ≥5), (e) score of JCS (0, >1), (f) family situation (living together or living alone), and (g) employment (employed or not employed). JCS: Japan Coma Scale; NIHSS: National Institutes of Health Stroke Scale

Cumulative driving resumption rates within 30 days were compared using the Kaplan-Meier method (Figure 2). The results showed a significantly higher driving resumption rate in participants aged <65 years than in those aged ≥65 years (p=0.013). Additionally, the NIHSS≥5 category had significantly higher rates of driving resumption than the 0 and 1-4 categories (p<0.0001). The driving resumption rates were significantly higher in patients diagnosed with ischemic stroke than in those diagnosed with cerebral hemorrhage (p=0.003). Additionally, the resumption rate was higher in the JCS=0 category than in the JCS≥1 category (p<0.0001), in patients living with family than in those living alone (p=0.033), and in those with employed work compared to those not employed (p=0.0001). No significant differences were found regarding sex (p=0.9).

Univariate Cox proportional hazards analysis revealed that patients with cerebral hemorrhage had a hazard ratio (HR) of 0.40 (95% confidence interval (CI): 0.21-0.75, p=0.002; Table 2). Patients aged ≥65 years had an HR of 0.53 (95% CI: 0.32-0.89, p=0.013). Those with a JCS score ≥1 had an HR of 0.08 (95% CI: 0.03-0.22, p<0.001), and those living alone had an HR of 0.44 (95% CI: 0.20-0.95, p=0.021). All these factors were significantly associated with a lower rate of driving resumption. In contrast, the HR for paid employment was 1.92 (95% CI: 1.08-3.41, p=0.019) and was significantly associated with a higher rate.

**Table 2:** Univariate and multivariate Cox proportional hazards model for resuming car driving within 30 days from stroke onset. ^1^HR: hazard ratio CI: confidence interval; NIHSS: National Institutes of Health Stroke Scale; JCS: Japan Coma Scale

	Univariable				Multivariable		
Variable	N	HR^1^	95% CI^1^	P-value	HR^1^	95% CI^1^	P-value
Sex	239			>0.9			0.4
Male		1.0	Reference		1.0	Reference	
Female		0.98	0.56, 1.70		0.76	0.43, 1.36	
Diagnosis	239			0.002			0.3
Brain hemorrhage		1.0	Reference		1.0	Reference	
Brain infarction		2.49	1.33, 4.67		1.39	0.69, 2.78	
Age	239			0.013			0.009
≤65 years		1.0	Reference		1.0	Reference	
>65 years		0.53	0.32, 0.89		0.46	0.25, 0.84	
NIHSS	239			<0.001			0.008
0		1.0	Reference		1.0	Reference	
1-4		0.77	0.40, 1.49		0.45	0.22, 0.92	
>5		0.13	0.05, 0.32		0.22	0.08, 0.56	
JCS	239			<0.001			<0.001
0		1.0	Reference		1.0	Reference	
≥1		0.08	0.03, 0.22		0.13	0.04, 0.37	
Family situation	239			0.021			0.12
Living alone		1.0	Reference		1.0	Reference	
Living together		2.30	1.05, 5.03		1.82	0.81, 4.11	
Employment	239			0.019			0.5
Employed		1.0	Reference		1.0	Reference	
Not employed		0.52	0.29, 0.93		0.80	0.40, 1.61	

Multivariate Cox proportional hazards analysis revealed an HR of 0.46 (95% CI: 0.25-0.84, p=0.009) for individuals aged ≥65 years, 0.22 (95% CI: 0.08-0.56, p=0.008) for those with an NIHSS score ≥5, and 0.13 (95% CI: 0.04-0.37, p<0.001) for those with a JCS≥1, all associated with a lower rate of driving resumption.

## Discussion

We aimed to investigate whether specific neurological and social background factors at the time of stroke onset were linked to the occurrence of driving resumption events within 30 days of onset in patients hospitalized due to a first-ever ischemic stroke or hemorrhagic stroke. The results showed that younger age (<65 years), mild NIHSS score (<5 points) at transport, and JCS of 0 (clear consciousness) at transport were associated with early driving resumption.

It was easier for younger participants aged <65 years to resume driving compared to those aged ≥65 years. Older patients generally report slower recovery after stroke than younger patients [23,24]. In addition, driving behavior has been reported to decline with age in terms of perceptual, motor, and cognitive functions [25]. For instance, the operation of the steering wheel may become awkward, acceleration and braking may slow down, and individuals may miss people, signs, and signals owing to decreased visual acuity and narrowing of the visual field. Additionally, decreased attentional function may render individuals unable to pay attention to their surroundings. In addition, younger patients showed a higher ability to adapt to neuropsychological tests [26] and DS and were considered to benefit more from driving rehabilitation [27]. Therefore, age-related factors affecting post-stroke recovery, as well as driving-related functions and abilities, may have influenced the resumption of driving within 30 days after stroke.

Lower NIHSS scores at the time of transport, indicating milder neurological damage, were associated with a quicker resumption of driving. Previous studies have shown that the NIHSS score is a reliable predictor of return to work [28], functional outcomes [29], and discharge destinations [30] in patients with stroke. Specifically, if the NIHSS score is <5 points, approximately 80% of patients are predicted to be dischargeable [31,32]. Therefore, patients expected to be discharged home are likely to consider resuming driving when they return to the community.

Patients with a JCS score of 0 found it easier to resume driving than those with a JCS score ≥1. Shigematsu et al. [33] reported that 64% of patients with stroke and a JCS score of 0 at onset did not have any symptoms or significant disability and were able to perform normal activities without assistance 30 days after onset. While GCS is widely used internationally for assessing disturbances in consciousness, the JCS demonstrates greater sensitivity in detecting near-normal states [34]. These findings suggest that even with a JCS score of 1, instrumental activities of daily living (IADLs) such as driving may be impaired. Furthermore, a GCS score of 15, indicating the mildest impairment, includes a mixture of JCS scores of 1 and 0, which may complicate accurate predictions. Therefore, it is important to use the JCS as an index for determining when to resume driving in the acute post-stroke period. A low JCS score at the time of transport is expected to be beneficial for the ability to resume driving within 30 days.

In our study population, the number of male participants was higher than that of female participants; however, no significant effect of sex on driving resumption rates was observed. In Japan, while the proportion of women holding driving licenses has increased in recent years, men still constitute a greater number of driving license holders [35]. This observation is consistent with findings by Ouellet et al. [17] who also reported a higher proportion of men. Therefore, our study population reflects the current situation in Japan and is supported by previous research. Moreover, there is a lack of literature reporting a direct effect of sex differences on driving resumption rates. Perrier et al. [36] reported that being female might indirectly reduce driving resumption rates one year after stroke through mediating factors such as fatigue, strength, and motor activity. As our study focuses on driving resumption within 30 days of stroke onset, the influence of sex on driving resumption may not be as pronounced as observed in studies examining driving resumption one year post-stroke. Additionally, individuals who consider resuming driving within 30 days are likely to have milder stroke symptoms, potentially reducing their susceptibility to sex-related effects. Furthermore, although sex may influence social background factors, our study found no association between sex and driving resumption rates, even after adjusting for factors such as family situation and employment. Considering these factors, it is likely that the impact of sex on driving resumption within 30 days post-stroke is minimal or negligible.

Furthermore, this lack of association was found in the results of this study also for social background factors such as living arrangements or employment status. While such factors may not significantly affect diagnosis, or evaluation and training by occupational therapists and others, social background may contribute to make patients feel compelled to resume driving within one month of the onset of illness despite guidelines prohibiting it, as previously reported [16]. We found no significant association between social background and driving resumption within 30 days at the population level. However, at the individual level, some patients resumed driving out of necessity despite being prohibited from doing so by their doctors or license centers. It is possible that such cases may have been relatively rare in the study population and, therefore, may not have reached significance during the analysis.

This study has some limitations. First, it is based on a single-center database, which may limit its generalizability. Second, we excluded patients with unknown outcomes and for whom time information was unknown. Reportedly, some patients in acute-care hospitals lack documented medical records related to driving [17]. In this study, some patients expressed a desire to resume driving, but their outcome and time information were unknown. Including these patients in the analysis could have led to different results. Third, some patients who were mildly ill but were transferred to a different hospital due to a diagnosis of another disease or condition during their hospitalization were not evaluated. Additionally, the families of some patients strongly opposed the resumption of driving. It is possible that unexamined background factors beyond those considered in this study may be relevant to the resumption of driving. Fourth, in this study, neuropsychological tests and other assessment items typically used during driving evaluations were not selected as factors. This decision was based on the potential for significant variation in the timing of driving evaluations depending on the severity of the condition. For instance, mild cases might be evaluated within 30 days, while severe cases might be evaluated after more than 30 days. This could be an unmeasured factor. Additionally, selecting assessments used to determine directly the resumption of driving could compromise the independence between the outcomes and the factors. Fifth, the resumption of driving not a little depend on the patient's willingness and permission from the attending physician. Finally, we did not adequately assess the validity of resuming driving, such as car accident after resuming driving. However, to the best of our knowledge, this study is the first to identify factors during ICU admission that are associated with the resumption of driving within 30 days. A significant strength of this research is the use of data from the JSDB, a representative dataset for stroke in Japan.

## Conclusions

We conducted a time-to-event analysis to examine the association between neurological and social background factors at the time of transport and the resumption of driving within 30 days of onset in patients with a first acute ischemic stroke or cerebral hemorrhage. The findings indicated that patients who were older than 65 years and had an NIHSS score higher than 5 and a JCS score higher than 1 exhibited a lower rate of driving resumption. These findings indicate that the information gathered at ICU admission can help identify individuals who may be able to resume driving within 30 days. This can aid with the provision of support and education to facilitate the efficient resumption of driving after an acute event.
